# Clinical Notes from Vienna

**Published:** 1886-10

**Authors:** W. S. Caldwell

**Affiliations:** Vienna


### FOREIGN CORRESPONDENCE.

CLINICAL NOTES FROM VIENNA.

*To the Editors of the* CHICAGO MEDICAL JOURNAL AND EXAMINER.

*Gentlemen:*

In my last letter to the JOURNAL AND EXAMINER, I promised to give, in the next, some surgical notes, taken at the clinic of Professor Ghiersch, in Leipzig; but the material from which to draw has so increased on my hands, that I have concluded to give notes of a single case only, from that clinic, and to draw also from the lectures of Professor C. Braun. The patient was a woman 68 years old, thin in flesh, but prior to the present attack, four months ago, in tolerably good health.

At that date she began to complain of a burning sensation in her left foot and toes. Soon after, a blue spot appeared on the top of the great toe, which became extremely painful, then turned black. The same process rapidly extended upward until the foot was gangrenous as far as the ankle-joint. Professor Ghiersch gave a short *résumé* of the pathology of this form of gangrene, saying that it was brought on by an atheromatous condition of the blood-vessels, which, as they approached the lower extremities, become smaller and more easily obliterated. The force then required to carry on the circulation being greater, the two combined influences rendered the feet and toes eminently predisposed to this disease. As an application, he used two parts of starch to one of iodoform, sprinkled over the surface, to which he applied cloths wet in one part of alcohol, two parts of water and (five per cent. of the whole) carbolic acid. He reviewed the line of treatment formerly pursued, that is, to let the parts alone until a perfect line of demarcation was formed by nature. "By that time," he remarked, "your patient will be worn out."

Then, again, the nearer you can get to the centre of the circulation, the more potent will be the heart's action to carry it on; so, in this case, instead of waiting for a line of demarcation between sound and diseased tissues, he amputated at the union of the lower and middle third of the femur.

I was much interested in this old lady's case, and a physician wrote me a month later that the patient had entirely recovered.

June 1, 1886. The first patient presented today was a woman, 47 years old, from whom Professor Braun had removed with the galvano-cautery the entire cervical portion of the womb, four years ago, on account of cancerous infiltration. The patient rapidly improved, and everything went on well until four months ago, when she began to suffer from dysmenorrhœa, and for the last two months her catamenia have failed entirely to appear. Above the *symphysis pubis* a tumor could be felt as large as a small cocoanut. An attempt to introduce a sound into the womb entirely failed. There was no cervix, as that had been cut away. A small depressed point in the centre of the cicatricial tissue showed where the uterine orifice had previously existed, but which was now completely obliterated. A long, narrow bistoury was plunged in at this point as nearly as possible in a line with the axis of the tumor that was to be felt above the pubis. A large quantity of dark grumous blood followed this incision. A sound was now introduced into the womb to a depth of six inches. The opening was enlarged by incisions made antero-posteriorly and laterally; the womb was well washed out with a 1 to 1000 solution of thymol; an iodoform suppository was introduced into the uterine cavity; the vaginal vault was sprinkled over with iodoform; the vagina was tamponed with iodoform gauze and the patient was put to bed. Professor Braun remarked, in connection with this case, that all these antiseptic precautions were absolutely necessary, as it was in just this class of cases that we might expect putrid changes to occur in the uterine cavity unless these precautionary measures were faithfully carried out. He then gave a general outline of his very extensive experience in the management of cancer of the womb. His deductions led him to lay down the following general rules:

1st.—That the *total* extirpation of the womb for malignant disease had proved a failure; that if the cancerous infiltration had extended so far that one could not remove it by the removal of the cervix, on account of the great vascularity of the parts and their numerous lymphatic glands, one might be assured that even after extirpation of the entire womb there would still remain behind in its connective tissue the nidus from which would soon spring up new cancerous nodules that would destroy the patient.

2d.—For the removal of the cervical portion of the womb he prefers the galvano-cautery. While it may be followed occasionally by the same accident that befell this patient, viz., a cicatricial closure of the *os uteri*, the disease is less liable to recur when thus operated upon than when the knife is used.

He cited in this connection the history of several patients upon whom he had operated a quarter of a century ago and who were still alive and well. This brought to my mind a lecture I heard him deliver eight years ago, in which he dwelt at length on the difficulties connected with an *early* diagnosis of cancer of the cervix. He said at that time that he was in the habit of cutting out a piece of the suspected tissue and sending it to a microscopist for his opinion. Not being always satisfied with the diagnoses given, he took to sending specimens to two different men, and often got two opinions; so that he had lost faith even in the infallibility of the microscope, and he believed that there were a large number of cases in which a positive diagnosis was impossible in the *earlier* stages of the disease.

The next patient was a woman who had just entered the hospital and who had miscarried at her own house ten days before, in the third month of pregnancy. Two days after, flooding began and she lost a large amount of blood. She was put in Sims' position, a speculum was introduced, the posterior lip of the womb was caught with Bozeman's forceps and brought well down. A large semi-blunt curette was introduced into the womb and the after-birth scraped away. Professor Braun prefers this mode of operating to the introduction of the finger, as he claims one can operate through a narrower os, and do less violence to the womb. The womb was washed out with thymol, as before described; a suppository of iodoform was placed within the os; the vagina was tamponed with iodoform gauze and the patient was sent to bed. This tampon is usually removed at the end of twenty-four hours, and if the patient has no increase of temperature no other treatment is instituted.

The next patient was one who had been delivered four days before at full term, and the labour was natural. The after-birth followed soon after the child. Twelve hours ago she had a severe chill and her temperature was now 40° C. She was placed in Sims' position; a speculum was introduced; the anterior lip of the womb was caught with a tenaculum, a large blunt curette introduced and a considerable portion of the chorion curetted away. He dwelt at length upon the proper manner of performing this operation.

The curette is first carried well up to the fundus, and is then withdrawn with one long sweep until it emerges from the external os. In this way one is enabled to determine at what portion of the uterine cavity any abnormal substance may be located, and so know where to direct efforts for its removal. This woman's womb was washed out with a solution of bichloride of mercury, 1 to 4000, followed immediately by the thymol solution, 1 to 1000.

The iodoform suppository was used as before described; the vagina was tamponed with iodoform gauze and the woman sent to her bed. Professor Braun predicted that within eighteen hours this woman's temperature would be normal, and that she would make a good recovery. In speaking of the use of intra-uterine injections in general, after childbirth or miscarriage, Professor Braun said that where he used them as a precautionary measure simply, that is after all operative procedures that involved the introduction of the hand or any instrument into the womb, he washed out the uterine cavity with thymol.

On the other hand, if he has to do with a case where there was a decided increase of temperature, showing that a septic process was already beginning, the thymol was too weak an agent to depend on as a destroyer of the existing germs, he uses first the stronger remedy, 1 to 4000 of the bichloride of mercury, and then follows it with the thymol to wash away the mercury to prevent *its* poisoning the patient. He said: "We have used this treatment in this hospital eighty-four times in the last six months, and while many condemn it altogether, or only use it with great reserve, I only regret that I have not used it earlier and oftener. The only patient we have lost in this hospital during that time from a puerperal process, I believe, died because we postponed our intra-uterine treatment too long."

"When I look over an experience of thirty years and remember the number of valuable lives I have seen sacrificed to what we jumble together and call *child-bed fever*, and reflect also how worthless most of the remedies for the same have proved in my hands, I am like the drowning man, ready to catch at a straw."

Hence the great interest with which I have studied this subject of their local treatment by means of intra-uterine injections.

Of the men who shall read this article, how do three-fourths treat child-bed processes? Possibly they wash out the vagina themselves or very likely order some old lady to do it. For this purpose an old Davidson syringe is called into use which no process short of its entire destruction could render aseptic. With this a solution of carbolic acid of very indefinite strength is used, generally with the solution a considerable quantity of air is thrown into the genital tract.

But their great agent in these crises is *quinine*. Though such men as Credé, and in fact a majority of the best observers here in Germany, think it of no use, in America we are now suffering from a quinine craze and we use it for everything. Or perhaps, following the directions of Professor Fordyce Barker, they put their patient on large doses of opium and an arterial sedative under the impression that they have as a part of the morbid process either a general or circumscribed peritonitis.

This part of their treatment, though rational enough in its place, can only fulfill one or two indications, viz., to relieve pain and lessen the peristaltic action of the bowels, and thus put the peritoneum at rest.

But in the matter of a more remote cause, where does this peritonitis come from? From a morbid process going out either from the vagina or uterus.

Is it not, then, at least a rational idea, when a patient first begins to manifest symptoms of disease, to treat locally the *centre* from which an endless chain of morbid processes may spread to the remotest parts of the system. It is to the *abuse* of the local treatment of the womb in the child-bearing woman that we probably owe a large part of the opposition to this measure.

Some twelve years ago Professor Winckel and other believers in the germ-theory, as a cause of most morbid processes, conceived the idea that if every woman's womb after confinement were washed out with a disinfecting solution, all puerperal diseases would be prevented. Every woman who bore a child was subjected to this operation. Midwives were instructed in the procedure and *Gebärmutterausspülungen* became the rage.

Experience soon demonstrated the fact that this treatment was being sadly overdone; in fact, that its misuse was worse than its entire omission. The practical question for us to consider is this: Under what circumstances should we use intra-uterine injections after child-birth? By a careful examination of the German literature of this subject during the last five years, including experiments made in the management of over 7,000 cases in the obstetrical wards of Professor Breisky, at Prague, the following deductions can be pretty safely depended upon, or at least they are the rules that now guide a majority of the men, who have charge of the different lying-in hospitals of Germany. First, vaginal injections should be used in every case *previous* to the birth of the child if the woman has a decided increase of temperature. After the birth of the child the *womb* itself should be irrigated. In the "*toucher Curs*" in Carl Braun's wards, where four men examine each patient, vaginal injections are used if the woman be taken in labour within twenty-four hours after the last examination. No uterine injection is used if she has no increase of temperature. Thorough uterine irrigation should be used (one to three quarts of the fluid) in every case where an operative procedure is found necessary, whether that be the introduction of the hand or any instrument into the uterine cavity. It should be used in every case where a considerable increase of temperature occurs, unless such increase can be traced directly and positively to some cause outside of the genital tract.

These injections should be used only with an irrigator, and should be used *promptly;* for after the patient has become contaminated by the transmission of the effete matter to distant parts of body, local treatment will be of no avail. The only case of child-bed fever lost in Carl Braun's wards during the last school year was one in which the injection was too long delayed.

The next point is, how are they to be used?

Dr. Fischel, who reports Professor Breisky's cases, in a long article in the 20th volume of the "*Archiv. für Gynækologie*," says that one of the greatest dangers in the use of an intra-uterine injection is the liability to infect the womb by carrying poisonous matter with the tube from the vagina into the uterine cavity. The first step in the operation, he says, is to thoroughly disinfect the *vagina* by washing it out with a 5 per centum solution of carbolic acid. He lays it down as a fundamental fact that one cannot tell from what point either in the vagina or the womb it is from which the poisonous matters are being absorbed which are producing the mischief.

A few wounds are likely to exist at both points. To protect these wounded surfaces from infection, nature pours out upon their surfaces a layer of plastic lymph, so that effete matters are not easily absorbed; and if this were not the case, a majority of women would die in child-bed. Hence he says that very often a woman is doing well and a vaginal injection is used for cleanliness and at once she is taken with a chill and has a high fever for a time. This is because there has been washed away the dam that has protected her from infection.

It follows therefore that when injections, either vaginal or uterine, are used they must be used with great *thoroughness.* After the vagina is thoroughly washed out, Breisky examines its surface very carefully and touches all denuded points with the tincture of iodine; though Carl Braun does not use this.

The idea is that by this means there is left a surface more healthy as well as less liable to absorb the foul secretions from the womb and vagina.

The author dwells at some length on the subject of permanent irrigation of the vagina and womb, and, after a long trial of the same, has abandoned its use.

I find myself in a field so wide that its full survey is impossible in the space to be occupied in the JOURNAL AND EXAMINER, and I will close with a few words as to the agents best to be used in uterine injections. Braun's ideas on this subject can be gleaned from what I have already written, except that he considers any solution of carbolic acid stronger than two per centum a dangerous remedy if the woman has had a child at full term.

On the other hand, Breisky uses the carbolic acid solution of five per centum strength. This he uses at first, and after that a solution of chloride of lime.

Under no circumstances is the use of these remedies to be entrusted to a midwife or a nurse.

Glass tubes long and curved are the kind used here to irrigate the womb at full term, as they can be easily disinfected. In the lying-in wards here in Vienna the irrigators are also made of glass. The injections are not warmed but used at the temperature of the room.

Where injections are used in cases of miscarriage, where of course the os is comparatively narrow, Bozeman's or Dr. Breus's intra-uterine syringes are used. If the womb in the latter class of cases needs to be dilated to get away an afterbirth, Braun prefers decidedly the Hegar dilator to the sponge or other forms of tent. The Barnes dilator, he remarked, is a nice thing to keep in your case, but it will always fail you when you want to use it.

Very truly yours,

W. S. CALDWELL.

Vienna, August 11, 1886.

